# Risk of dementia or cognitive impairment in non-alcoholic fatty liver disease: A systematic review and meta-analysis

**DOI:** 10.3389/fnagi.2022.985109

**Published:** 2022-09-20

**Authors:** Luping Wang, Bowen Sang, Zuyan Zheng

**Affiliations:** ^1^Department of Acupuncture and Massage, Heilongjiang University of Traditional Chinese Medicine, Harbin, China; ^2^Department of Acupuncture, The First Affiliated Hospital of Heilongjiang University of Chinese Medicine, Harbin, China

**Keywords:** non-alcoholic fatty liver disease, NAFLD, dementia, vascular dementia, Alzheimer's disease, cognitive impairment, meta-analysis, systematic review

## Abstract

**Objectives:**

To investigate whether non-alcoholic fatty liver disease (NAFLD) increases the risk of dementia or cognitive impairment.

**Methods:**

A systematic search of the literature in the PubMed, Excerpta Medica Database (EMBASE), Cochrane Library, and Web of Science databases was conducted, covering the period from the inception of each database to 22 May 2022. Statistical analysis of non-alcoholic fatty liver disease and the risk of cognitive impairment or dementia based on data extracted from each article was performed using Stata software v. 16.0. The quality of this study was assessed using the Newcastle-Ottawa Scale (NOS) for assessing the quality of cohort and case-control studies and the American Agency for Healthcare Research and Quality (AHRQ) methodology checklist for assessing the quality of cross-sectional studies. Funnel plots and the Egger's test were used to assess publication bias.

**Results:**

We included 7 studies comprising 891,562 individuals from 6 countries, which were published between 2020 and 2022. The pooling analysis showed that a history of NAFLD was associated with cognitive impairment [odds ratio (OR) = 1.44; 95% CI: 1.17–1.78; heterogeneity (*I*^2^) = 0%; *P* = 0.001]. A history of NAFLD was not associated with an increased risk of all-cause dementia (OR = 1.03; 95% CI: 0.97–1.09; *I*^2^ = 84.7%; *P* = 0.341) or Alzheimer disease (OR = 0.95; 95% CI: 0.83–1.09; *I*^2^ = 61.0%; *P* = 0.489). In contrast, NAFLD was associated with an obvious reduction of the risk of vascular dementia (OR = 0.88; 95% CI: 0.79–0.98; *I*^2^ = 0.0%; *P* = 0.020). In the subgroup analysis, male and female patients with NAFLD showed an equal risk of dementia or cognitive impairment. The risk of dementia or cognitive impairment in the cross-sectional study (OR = 1.49; 95% CI: 1.19–1.88; *I*^2^ = 0.0%; *P* = 0.001) was slightly higher than that in the retrospective cohort (OR = 1.03; 95% CI: 0.97–1.09; *I*^2^ = 84.3%; *P* = 0.294).

**Conclusions:**

NAFLD was associated with an increased risk of cognitive impairment and a decreased risk of vascular dementia. More studies are needed to clarify the pathophysiological mechanism underlying the association between NAFLD and dementia or cognitive impairment.

**Systematic review registration:**

https://www.crd.york.ac.uk/prospero/#recordDetails, identifier: CRD42022334492.

## Introduction

Non-alcoholic fatty liver disease (NAFLD), defined by the finding of hepatic steatosis on imaging or histology after exclusion of alcohol and other definite factors as causes for liver damage (Tokushige et al., 2021), has become one of the most prevalent causes of chronic liver disease worldwide (Younossi et al., 2016). Among waitlist registrants, NAFLD is now the fastest-growing indication for liver transplantation (Wong et al., 2015). In Australia, NAFLD has accounted for a large and expanding proportion of liver disease burden, with Markov models predicting a 23% increase in NAFLD cases from 2019 to 2030 (Adams et al., 2020). The burden of NAFLD also continues to rise significantly in China and Japan, with the highest growth occurring in China because of urbanization, and the lowest growth occurring in Japan as a result of a shrinking population (Estes et al., 2018). NAFLD is associated with a range of common chronic disease risk factors and poor lifestyle habits, such as hypertension, obesity, visceral fat accumulation, smoking, staying up late, and drinking (Mirmiran et al., 2017; Åberg, 2020; Adams et al., 2020). The data from current research indicate that NAFLD patients may have early or subtle cognitive dysfunction, including in the visuospatial and executive function domains (Celikbilek et al., 2018). Consequently, attention should be paid to the cognitive function of patients with NAFLD.

Dementia, which afflicts 50 million individuals worldwide (2019), results in a significant decrease in an individual's cognitive level and is accompanied by memory and behavior-related disorders resulting from brain damage due to injury or illness (Lee et al., 2022). Data from 2015 showed that there were an estimated 47 million dementia cases worldwide, costing an estimated $818 billion (Xiang et al., 2021). With an aging population and a lack of effective preventive measures, the number of people living with dementia worldwide will climb to 131.5 million by 2050 (Prince et al., 2015). Coincidently, researchers have found that both NAFLD and dementia share 2 important biological risk factors, apolipoprotein E (APO-E) (Yang et al., 2005; Rasmussen, 2016) and adiponectin (ADPN) (Buechler et al., 2011; Rizzo et al., 2020). There is increasing evidence that NAFLD patients are at an increased risk of developing hypertension (Ciardullo et al., 2022), depression (Gu et al., 2022), and type 1 diabetes mellitus (De Vries et al., 2020).

Previous studies have found that NAFLD is associated with poorer cognitive function. Nonetheless, the studies conducted to date have been limited to an observational design and have demonstrated methodological heterogeneity, using various diagnostic tools to assess cognitive function (George et al., 2022). Meanwhile, systematic reviews and meta-analyses concerning the association between NAFLD and cognitive impairment or dementia are lacking. We therefore conducted this meta-analysis to systematically search existing population-based, longitudinal evidence in published research to determine the association between NAFLD and the risk of cognitive impairment or dementia.

## Method

This meta-analysis was conducted in accordance with the guidelines of the PRISMA (Preferred Reporting Items for Systematic Reviews and Meta-Analyses) (Page et al., 2021). The protocol was preregistered in the International Prospective Register of Systematic Reviews (PROSPERO) platform (registration no. CRD42022334492).

### Search strategy

The PubMed, EMBASE, Cochrane Library, and Web of Science databases were systematically searched from their inception to 22 May 2022. There were no language restrictions, and the search strategy combined the use of medical subject headings (MeSH) and keywords. The search terms in the study were as follows: (“Non-alcoholic Fatty Liver Disease ” OR “Non-alcoholic Fatty Liver Disease” OR “NAFLD” OR “Non-alcoholic Fatty Liver*” OR “Non-alcoholic Steatohepatiti*”) AND (“Dementia” OR “Alzheimer's disease” OR “Cognitive decline” OR “Cognitive impairment” OR “Cognitive disorder” OR “Cognitive dysfunction”). The complete search strategy is presented in Additional File 1. Additionally, we conducted manual searches of reference lists and related reviews of included studies for other relevant articles.

### Eligibility criteria

Trials were included according to the following eligibility criteria: (1) cohort or case–control study designs; (2) reports of dementia or cognitive impairment diagnosis as the outcome, with the diagnostic criteria of NAFLD being hepatic steatosis in addition to more than 1 of (i) overweight or obesity status, (ii) type 2 diabetes mellitus (T2DM), and (iii) metabolic dysregulation; and (3) investigations of the association of NAFLD with the risk of dementia or cognitive impairment. If multiple data were reported from the same study, we included the study with the longest follow-up or the largest number of participants.

### Exclusion criteria

The following literature was excluded: (1) conference abstracts or study protocols, (2) duplicate publications, and (3) studies with no outcomes or inadequate data.

### Research selection

The literature included in this study was independently screened by 2 reviewers (WLP and ZZY) based on eligibility and exclusion criteria. First, duplicate articles and those deemed to not be relevant to this study based on titles and abstracts were excluded. Thereafter, potential articles were identified, downloaded, and read in full to ensure that data from the article could be effectively included. In case of disagreement concerning the inclusion of an article, the 2 researchers discussed with a third reviewer to reach a consensus.

### Data extraction

According to guidelines on data extraction for systematic reviews and meta-analyses (Taylor et al., 2021), the 2 above-mentioned reviewers independently extracted and summarized the following information from each of the included studies: first author, year of publication, country, study type, sample size, study period, number of follow-up years, age of participants, gender, diagnosis of dementia or cognitive impairment, dementia type, and adjustment for confounders.

### Risk of bias assessment

We assessed the quality of the included articles using the Newcastle–Ottawa Scale (NOS) for quality assessment (Stang, 2010), focusing on 3 aspects: selection, comparability, and outcome. The score ranged from 0 to 9 points for cohort studies and case–control studies. According to the NOS, the higher the score is, the better the quality of the included studies. We considered the NOS scores of 0–3, 4–6, and ≥7 as indicative of low, medium, and high quality, respectively. The quality of a cross-sectional study was assessed using the American Agency for Health Care Research and Quality (AHRQ) methodology checklist. The checklist contains 11 items, with the risk of bias scored as 1 for “Yes” and 0 for “Unclear” or “No” risk. After recording the total score, the articles were divided into 3 grades: “low” (0–3 points), “medium” (4–7 points), and “high” (8–11 points).

### Statistical analysis

To assess the association between NAFLD and the risk of dementia or cognitive impairment, we extracted the adjusted odds ratio (OR) and 95% CI from each study. The extracted adjusted OR and 95% CI were statistically analyzed using Stata v. 16.0 software (Stata Corp, College Station, TX, USA). The heterogeneity of the included studies was assessed by the chi-square test and *I*^2^. When *P* was>0.10 for the χ2 test or when *I*^2^ was <50%, statistical heterogeneity was considered non-significant, and the fixed-effects model was subsequently adopted. If significant heterogeneity was indicated from *I*^2^ > 50% or *P* < 0.1, the random-effects model was employed. Robustness of the results with respect to the overall effect of statistics and sources of heterogeneity were verified through subgroup analysis and sensitivity analysis. Publication bias was confirmed by visual inspection of funnel plots, and the Egger regression test was used to statistically detect publication bias for asymmetry.

## Results

### Literature search

**Figure 1:**
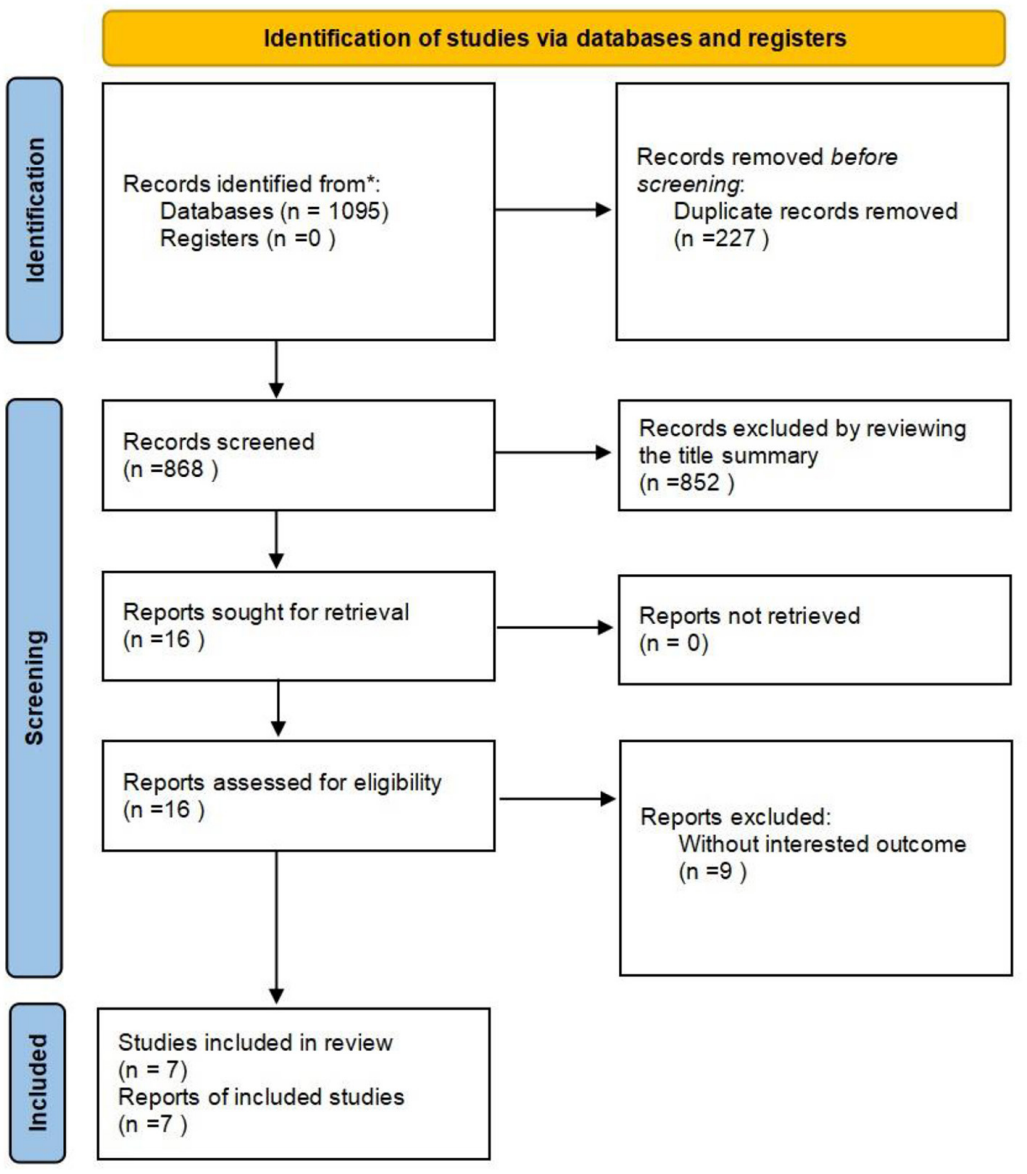
Studies screening process.

The systematic search of studies published before 22 May 2022, identified 1,095 results. Of the 1,095 articles, 227 were duplicate articles and were excluded. After screening the title and abstract, another 852 articles were excluded. After perusal of the full text of the remaining of the articles, 9 articles were excluded. Of these 9 articles, 8 failed to provide relevant data and outcomes and 1 article provided incomplete data. Ultimately, 7 studies (Labenz et al., 2021; Lampignano et al., 2021; Shang et al., 2021; Jeong et al., 2022; Kim et al., 2022; Liu et al., 2022; Yu et al., 2022) were included in this study. The search selection process is presented in Figure 1.

### Study characteristics

Seven studies were included in this review. Five studies (Labenz et al., 2021; Shang et al., 2021; Jeong et al., 2022; Kim et al., 2022; Liu et al., 2022) were cohort studies and 2 studies (Lampignano et al., 2021; Yu et al., 2022) were case–control studies. The 7 studies were published from 2020 to 2022 and involved a total of 891,562 individuals, with sample sizes ranging from 1542 to 608,994 individuals per study. The average follow-up time ranged from 3 to 9.5 years. There were 2 studies (Jeong et al., 2022; Kim et al., 2022) from Korea, 1 (Yu et al., 2022) from the United States, 1 (Liu et al., 2022) from China, 1 (Shang et al., 2021) from Switzerland, 1 (Labenz et al., 2021) from Germany, and 1 from (Lampignano et al., 2021) Italy. The results of 5 studies (Labenz et al., 2021; Lampignano et al., 2021; Shang et al., 2021; Jeong et al., 2022; Kim et al., 2022) described outcome measures of dementia, and the 2 other studies (Liu et al., 2022; Yu et al., 2022) involved cognitive impairment. The main characteristics of the included studies are shown in Table 1.

**Table 1:** Characteristics of studies included in the review.

**Authors**	**Year**	**Country**	**Dementia type/cognitive impairment**	**Sample size**	**Study period**	**Follow up years**	**Age (years)**	**Diagnosis of dementia /cognitive impairment**	**Confounders adjusted**	**AHRQ**
**cross-sectional study**	
Qiang Yu	2022	USA	Cognitive impairment	Total: 4,973 Non- NAFLD:3,333 NAFLD:1,640	1988–1994	/	Non- NAFLD:36.28 ± 10.43 NAFLD:39.10 ± 11.11	The serial digit learning test (SDLT), the simple reaction time test (SRTT) and the symbol digit substitution test (SDST)	sex, age, ethnicity, education level, and history of stroke	7
Luisa Lampignano	2021	Italy	All-cause dementia	Total: 1,542	2015–2018	3	over 70	The diagnosis of dementia was made according to the Diagnostic and Statistical Manual of Mental Disorders, Fourth Edition criteria (DSM-4).	age, sex, education, hypertension, diabetes mellitus, alcohol consumption, smoking habit, stroke, cholesterol, and Apo-E	6
**Authors**	**Year**	**Country**	**Dementia type/cognitive impairment**	**Sample size**	**Study period**	**Follow up years**	**Age (years)**	**Diagnosis of dementia/cognitive impairment**	**Confounders adjusted**	**NOS**
**Retrospective cohort**	
Chi Hyuk Oh	2022	Korea	All-cause dementia	Total: 4,031,948 Non- NAFLD: 742,934 NAFLD: 1,263,376 Intermediate: 2,025,638	2008–2018	9.5	Non- NAFLD:53.6 ± 8.4 NAFLD:53.7 ± 7. 8 Intermediate: 53.9 ± 7.9	ICD-10 (F00- F03 or G30- 32)	age, sex, income, disability, residence area, hypertension, total cholesterol, fasting blood glucose, systolic blood pressure and diastolic blood pressure	8
Christian Labenz	2020	Germany	All-cause dementia, Vascular dementia, Alzheimer's disease	Total: 44,634 Non- NAFLD:22,317 NAFLD:22,317	2000–2015	7.8	73.4 ± 5.9	ICD-10( F00-F03, G30)/ICD-10(F06.7)	/	7
Qi Liu	2021	China	Cognitive impairment	Total: 1,651 Non- NAFLD:856 NAFLD:795	2015–2019	4	53.4 ± 8.4	Mini-Mental State Examination (MMSE)	age, sex, carotid stenosis, educational levels, body mass index, hypertension, diabetes mellitus, and hyperlipidemia.	7
Ying Shang	2020	Switzerland	All-cause dementia, Alzheimer's disease	Matched cohort General population n = 6,436 NAFLD:656	1971–2009	/	Matched cohort General population n = 48.2 (±SD 13.7) NAFLD:48.4 (±SD 13.6)	ICD-8,ICD-9,ICD-10	age, sex and municipality	8
Seogsong Jeong	2022	Korea	All-cause dementia, Vascular dementia, Alzheimer's disease	Total: 608,994 Non- NAFLD:193,739 NAFLD:269,441 Intermediate:145,814	2009–2010	/	65 (62–69)	ICD-10 (F00, F01, F02, F03, G30),ICD-10 (F00 and G30), ICD-10 (F01)	age, sex, body mass index, household income, systolic blood pressure, and fasting serum glucose, smoking, moderate-to-vigorous physical activity, and Charlson comorbidity index	9

NAFLD, non-alcoholic fatty liver; AD, Alzheimer's disease; VD, Vascular dementia

### Risk-of-bias assessment

The quality of included studies was assessed by the NOS scale, and the results are shown in Table 1. The average score across the 7 studies was 7.8, indicating that the overall quality was high. Among them, 1 article (Jeong et al., 2022) scored as high as 9 points, and the remaining studies scored 7 points and 8 points. According to the AHRQ criteria, the scores for the 2 cross-sectional studies (Lampignano et al., 2021; Yu et al., 2022) were 6 and 7, respectively, and both were classified as being of moderate quality.

### NAFLD and risk of cognitive impairment

**Figure 2:**
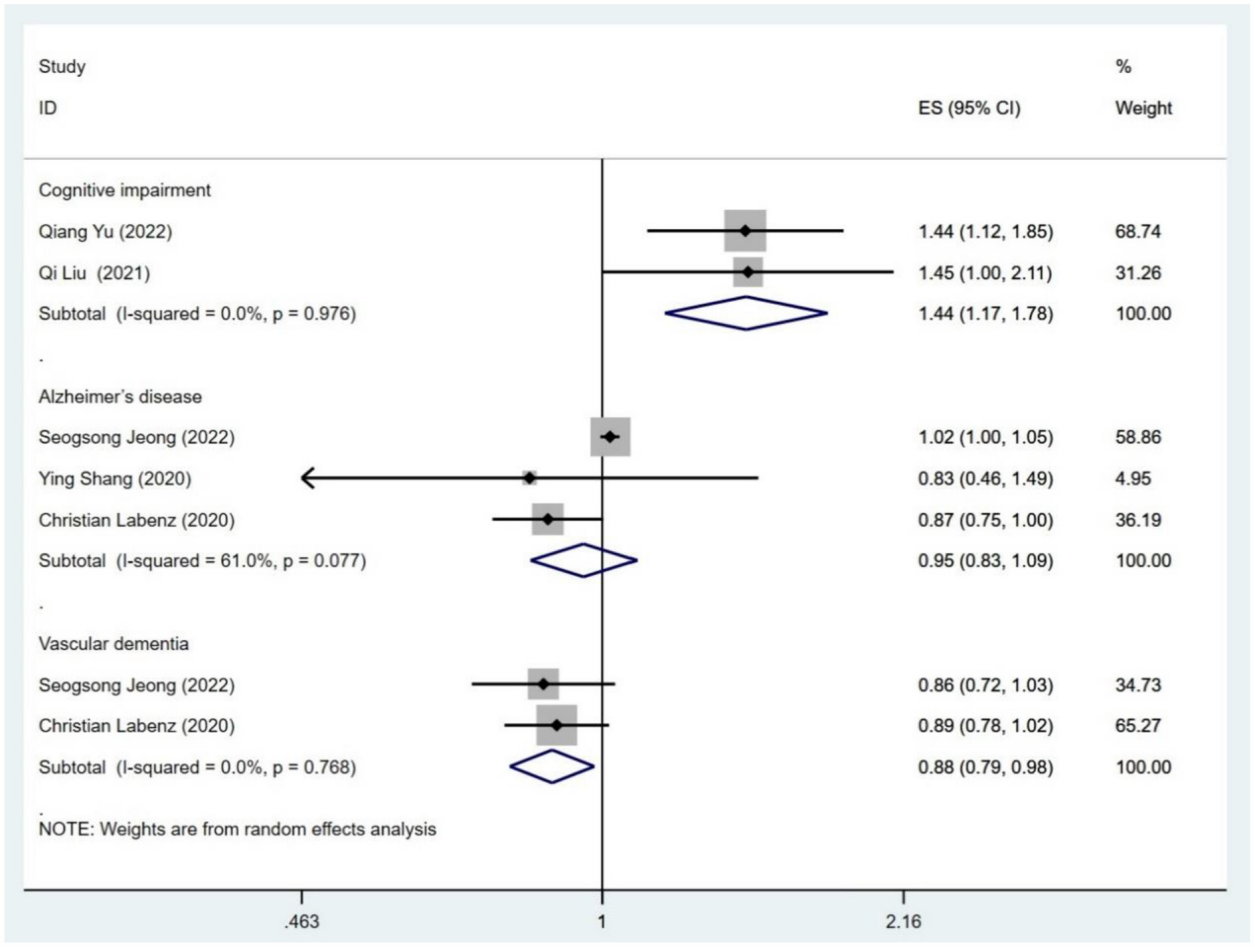
Meta-analysis of the risk of cognitive impairment, Alzheimer's disease and vascular dementia caused by NAFLD.

**Figure 3:**
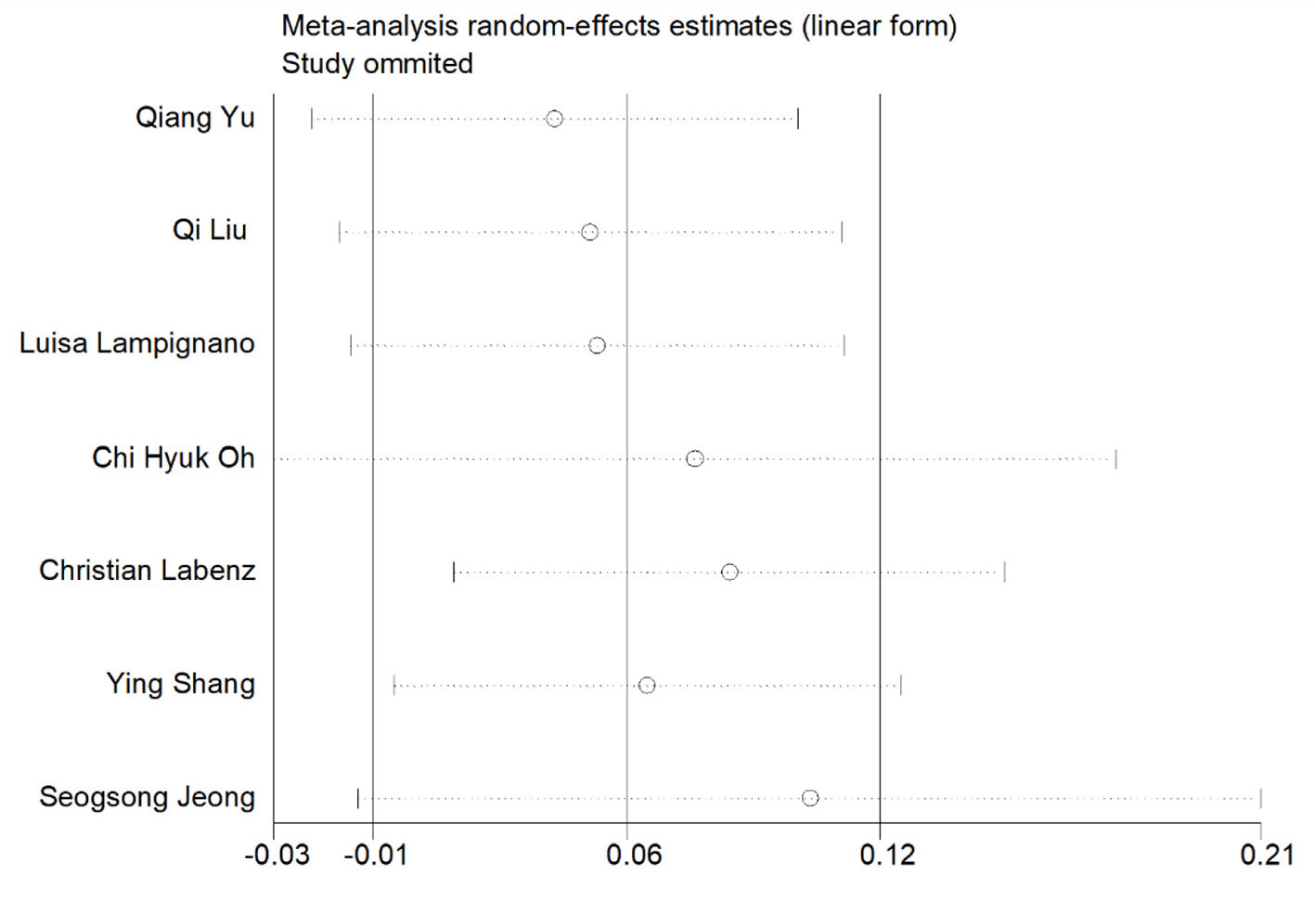
Sensitivity analysis of the risk of all-cause dementia and cognitive impairment caused by NAFLD.

Two studies (Liu et al., 2022; Yu et al., 2022) assessed the association between NAFLD and risk of cognitive impairment. As shown in Figure 2, data analysis showed a strong link between NAFLD and cognitive impairment (OR = 1.44; 95% CI: 1.17–1.78; *I*^2^ = 0%; *P* = 0.001). Through sensitivity analysis, the pooled-effect size was not reversed by individual studies, which means that the outcomes demonstrated robustness (Figure 3).

**Figure 4:**
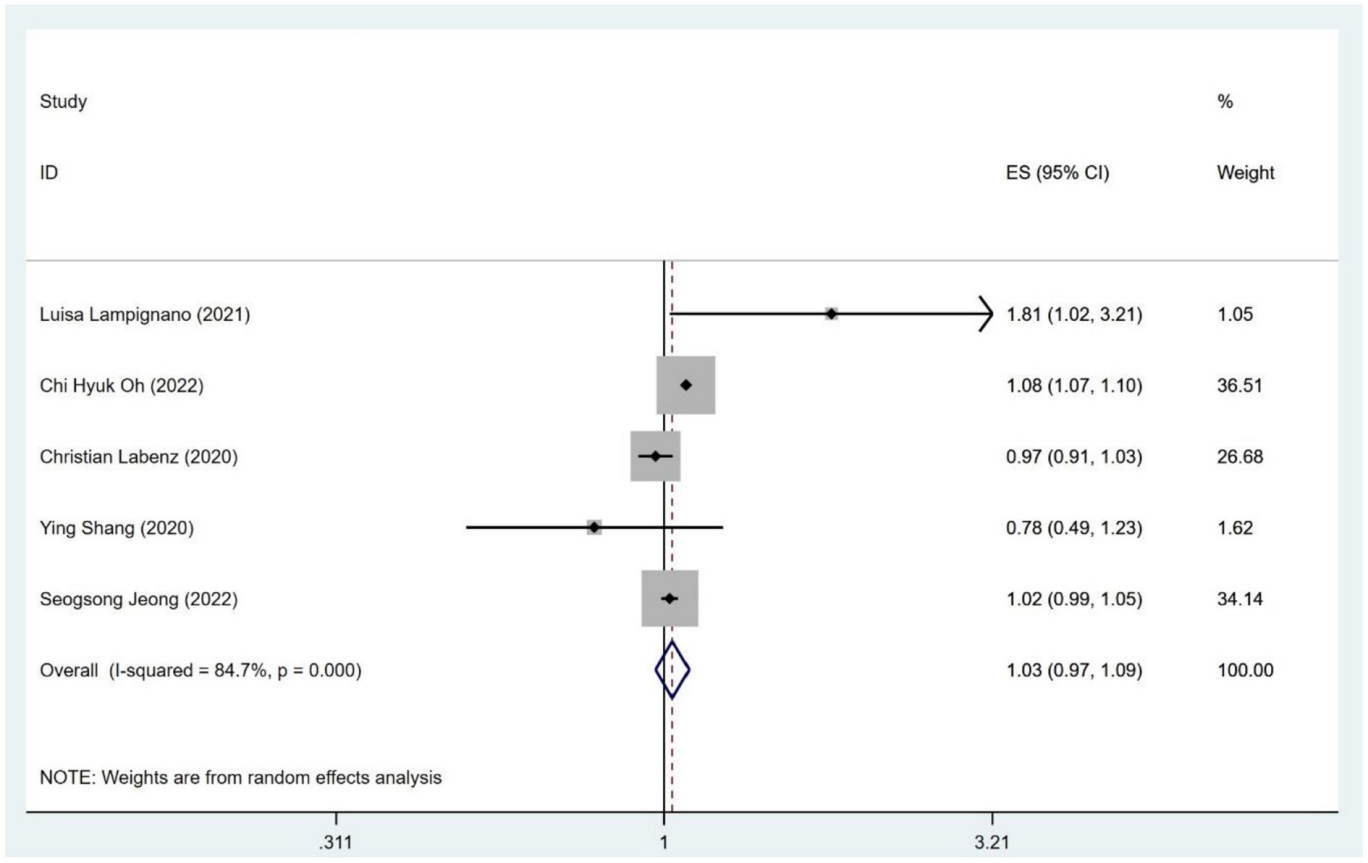
Meta-analysis of the risk of dementia caused by NAFLD.

**Figure 5:**
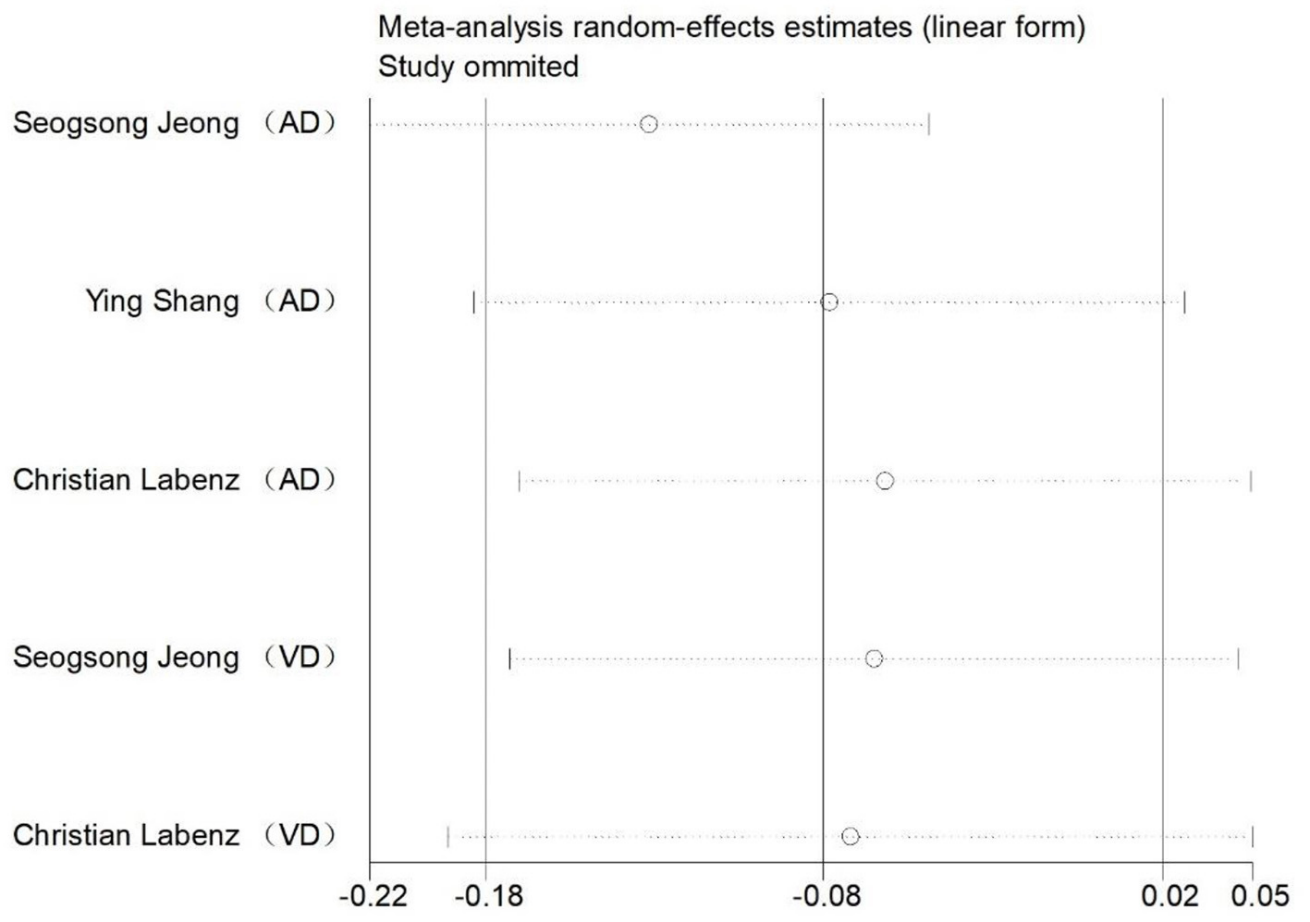
Sensitivity analysis of the risk of AD and VD caused by NAFLD.

As shown in Figures 4, 5 studies (Labenz et al., 2021; Lampignano et al., 2021; Shang et al., 2021; Jeong et al., 2022; Kim et al., 2022; Liu et al., 2022; Yu et al., 2022) described the association of all-cause dementia with NAFLD; however, results from the data showed that the relationship between NAFLD and an increased risk of all-cause dementia was not obvious (OR = 1.03; 95% CI: 0.97–1.09; *I*^2^ = 84.7%; *P* = 0.341). Since Alzheimer Disease (AD) and vascular dementia (VD) belong to the category of dementia, we extracted the data of AD and VD separately for reanalysis. Two of five studies (Labenz et al., 2021; Jeong et al., 2022) described the association between NAFLD and VD, with the data demonstrating a significant relationship (OR = 0.88; 95% CI: 0.79–0.98; *I*^2^ = 0.0%; *P* = 0.020; Figure 2). Three studies (Labenz et al., 2021; Shang et al., 2021; Jeong et al., 2022) evaluated the risk of AD in NAFLD and found that NAFLD was not associated with an increased risk of NAFLD (OR = 0.95; 95% CI: 0.83–1.09; *I*^2^ = 61.0%; *P* = 0.489; Figure 2). Through sensitivity analysis, the pooled-effect size was not reversed by individual studies, which means that the outcomes demonstrated robustness (Figure 5).

### Subgroup analysis

In a subgroup analysis of gender, 4 studies (Labenz et al., 2021; Shang et al., 2021; Kim et al., 2022; Liu et al., 2022) described the likelihood of dementia or cognitive impairment in females with NAFLD (OR = 1.09; 95% CI: 0.93–1.27; *I*^2^ = 83.2%; *P* = 0.273; Table 2) and 3 studies (Labenz et al., 2021; Kim et al., 2022; Liu et al., 2022) described the likelihood of dementia or cognitive impairment in males with NAFLD (OR = 1.01; 95% CI: 0.95–1.07; *I*^2^ = 31.0%; *P* = 0.765; Table 2). The data demonstrated that there was no gender difference in the chances of developing dementia in patients with NAFLD.

**Table 2:** Subgroup analysis for the risk of dementia or cognitive impairment in patients with NAFLD.

**Subgroups**	**Include study**	**OR (95% CI)**	**Heterogeneity**	
			*I*^2^ **(%)**	***P*** **-values**
**Study type**				
Cross-sectional study	2	1.49 (1.19–1.88)	0.00%	0.474
Retrospective cohort	5	1.03 (0.97–1.09)	84.30%	0.000
**Sex**				
Female	4	1.09 (0.93–1.27)	83.20%	0.000
Male	3	1.01 (0.95–1.07)	31.00%	0.235

In the subgroup analysis of study type, 2 studies (Lampignano et al., 2021; Yu et al., 2022) evaluated the risk of dementia or cognitive impairment using a cross-sectional study design and found that NAFLD was associated with an enhanced risk of dementia or cognitive impairment (OR = 1.49; 95% CI: 1.19–1.88; *I*^2^ = 0.0%; *P* = 0.001; Table 2). The other 5 included studies (Labenz et al., 2021; Shang et al., 2021; Jeong et al., 2022; Kim et al., 2022; Liu et al., 2022) showed that other retrospective cohorts of NAFLD were not associated with an increased risk of dementia or cognitive impairment (OR = 1.03; 95% CI: 0.97–1.09; *I*^2^ = 84.3%; *P* = 0.294; Table 2).

### Publication bias

**Figure 6:**
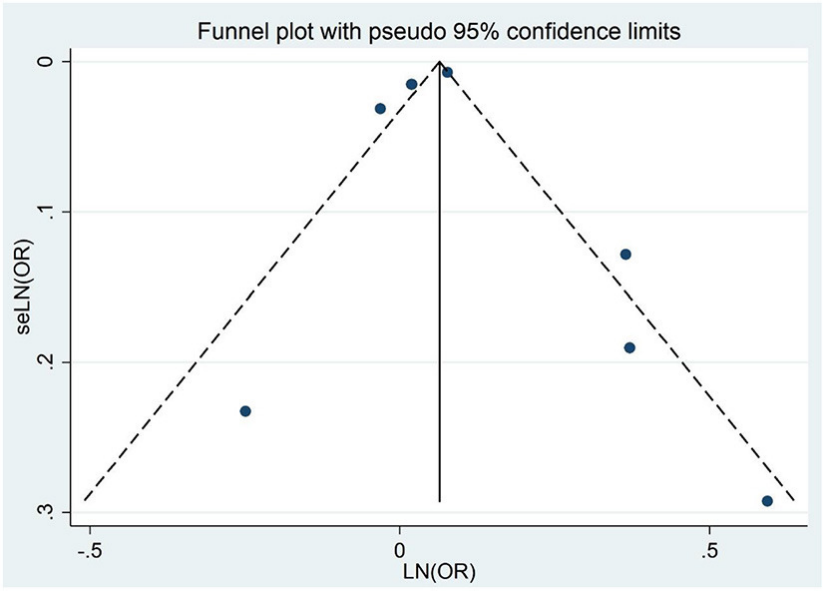
Figure showing the effect of NAFLD on all-cause dementia and cognitive impairment.

Through the visual inspection of the funnel plot, there was no evidence of a significant publication bias in the outcomes for NAFLD and the risk of dementia or cognitive impairment (Figure 6). Egger regression test (*P* = 0.952) also indicated no publication bias in our meta-analysis.

## Discussion

### Main findings

In this systematic review and meta-analysis of observational studies, a total of 7 population-based studies provided a comprehensive evaluation of the relationship between NAFLD and the risk of dementia or cognitive impairment in approximately 891,562 individuals. We found a significant increase in the risk of cognitive impairment among individuals with NAFLD, with an overall 1.44-fold increase in risk compared to healthy controls. In contrast, the association between NAFLD and VD was unexpected. Patients with NAFLD had a 0.88-times risk of developing VD; hence, patients with NAFLD were less likely to develop VD. Subgroup analyses showed that gender was not a significant factor in determining whether patients with NAFLD were at a high risk for dementia or cognitive impairment. In the subgroup analysis of study type, NAFLD was associated with a higher risk of dementia or cognitive impairment in cross-sectional studies compared with retrospective cohorts.

### Interpretation of findings

We found a statistically significant difference between NAFLD and cognitive impairment after undertaking separate statistical calculations for dementia and cognitive impairment although this result was based on just 2 observational studies. However, because of the low heterogeneity of the statistical results, this may increase statistical efficiency. The conclusions drawn from the results of the data can also be verified from the results of previous research. A review (George et al., 2022) of 11 observational studies showed NAFLD to be associated with low cognitive ability across several domains, such as “general cognition,” “mental speed, attention, and psychomotor speed,” and “ideas, abstraction, figural creations, and mental flexibility.” In the study by Sang et al. (Seo et al., 2016), 874 (19.5%) of 4,472 patients with confirmed NAFLD were diagnosed with cognitive impairment. However, the study failed to provide enough data to calculate the increased risk of cognitive impairment in relation to NAFLD. In addition, a prospective cross-sectional study (Filipović et al., 2018) suggested that a cognitive deficit was more pronounced in persons with NAFLD, and patients suffering from NAFLD had about a 4-times higher risk of having cognitive impairment as assessed by the Montreal Cognitive Assessment test. The study of Filipović et al. (2018) shows that the volume of gray and white matter in NAFLD patients with lower Montreal Cognitive Assessment (MoCA) scores may be significantly reduced. However, this study did not include data on the incidence of cognitive impairment due to NAFLD; hence, this result was not included in our meta-analysis. In contrast, more recent studies were added to the current analysis, thus providing robust evidence on the association between NAFLD and the risk of cognitive impairment.

After grouping by type of dementia, we found that NAFLD patients had a lower risk of VD than did patients with AD. The result of our meta-analysis showed that patients with NAFLD have a reduced risk of developing VD; however, converse, results were reported in a recent study by Wang et al. (2022), in which an overall 2.62-fold increase in the risk of VD among individuals with NAFLD was observed. Owing to the small number of the study populations included in this review and the fact that the control group of the study was not compared with healthy people, we were unable to further investigate the effect of NAFLD on dementia risk.

Subgroup analyses showed that there was no statistical interaction of NAFLD with male or female gender with respect to the likelihood of dementia or cognitive impairment, which is largely consistent with the results suggested by previous studies (Wang et al., 2022). In the subgroup analysis of study type, NAFLD was associated with an increased risk of dementia or cognitive dysfunction in cross-sectional studies compared to retrospective cohort studies. We speculate that this may be related to the following reasons: there were only 2 cross-sectional papers in the study, which might have reduced statistical efficiency, and the smaller sample size relative to the case–control study might influence the results.

The pathophysiological mechanisms of the association between NAFLD and dementia or cognitive impairment remain largely unclear. Nevertheless, some researchers continue to speculate upon the relationship between NAFLD and dementia or cognitive impairment. Systemic neuroinflammation has been considered to be one of the important causes of cognitive impairment and dementia in NAFLD. Liver-induced macrophages (Haukeland et al., 2006; Tosello-Trampont et al., 2012) infiltrated by lipid accumulation secrete cytokines and chemokines into the systemic circulation (Tosello-Trampont et al., 2012; Dixon et al., 2013; Kratz et al., 2014; Narayanan et al., 2016), but it is unclear how inflammation spreads across the blood–brain barrier to the brain (Viscogliosi et al., 2013; Miller and Spencer, 2014). In addition, cytokine-activated receptors on peripheral endothelial cells promote the release of proinflammatory factors in the central nervous system (Cai and Liu, 2012), thereby causing an inflammatory response in the brain to disrupt brain function. Second, studies have demonstrated that the dysfunction of the gut–liver–brain axis in patients with liver cirrhosis is associated with hepatic encephalopathy (Bajaj, 2014; Ahluwalia et al., 2016), which has led to an interest in determining whether or not cognitive impairment in patients with NAFLD is associated with altered gut microbiota. In the early stages of NAFLD, gut microbiota homeostasis is disrupted; for example, by small intestinal bacterial overgrowth and low-abundant bacteria (Bauer et al., 2002; Qin et al., 2014; Bajaj et al., 2015). These microbial signatures are the drivers of “leaky gut” (Miele et al., 2009; Li et al., 2013). Gut microbes that enter the systemic circulation, such as endotoxins, ammonia, and bacterial DNA, can initiate and propagate systemic and neuroinflammation leading to cognitive impairment or dementia (Bajaj, 2014; Liu et al., 2020). In addition, studies have found that vascular complications in patients with NAFLD (Targher et al., 2016), such as atherosclerosis and cerebrovascular dysfunction, may contribute to dementia or cognitive impairment (De La Torre, 2012; Long et al., 2015). The upregulation of cerebral blood volume was found to be significantly impaired by oxygenated hemoglobin concentration in patients with NAFLD as measured on a cognitively challenging verbal fluency task (Takahashi et al., 2017). Notably, in addition to the need for more research to further understand the pathological mechanism of this new metabolic encephalopathy caused by NAFLD, future research should pay increased attention to the accurate diagnosis of the disease and its impact on daily life, so as to formulate a better treatment plan for improving patients' quality of life.

### Implications and limitations

Our meta-analysis drew on existing evidence to summarize the association between a history of NAFLD and the risk of dementia or cognitive decline. Albeit, our results have some limitations. Due to the small amount of experimental data (acquired from all of the studies) and the methodological heterogeneity of the included studies, the accuracy of our results might have been affected. The diagnostic criteria for NAFLD and dementia or cognitive impairment are inconsistent, which might have biased the results. In addition, caution should be taken concerning the generalizability of the included studies' results. Since these studies were conducted in the United States, China, Italy, Korea, and Switzerland, regional bias may be present.

## Conclusion

This meta-analysis indicated that NAFLD is associated with an increased risk of cognitive impairment. In contrast, patients with NAFLD have a lower risk of VD. However, more studies are needed to clarify the pathophysiological mechanisms underlying the association between NAFLD and dementia or cognitive impairment.

## Data availability statement

The original contributions presented in the study are included in the article/Supplementary material, further inquiries can be directed to the corresponding author/s.

## Author contributions

LW would answer for design and conception of the article and drafted the manuscript. BS would answer for the collection and assembly of materials. LW and BS would answer for data interpretation and analysis. ZZ revised it. All authors reviewed and approved the final version of the manuscript.

## Conflict of interest

The authors declare that the research was conducted in the absence of any commercial or financial relationships that could be construed as a potential conflict of interest.

## Publisher's note

All claims expressed in this article are solely those of the authors and do not necessarily represent those of their affiliated organizations, or those of the publisher, the editors and the reviewers. Any product that may be evaluated in this article, or claim that may be made by its manufacturer, is not guaranteed or endorsed by the publisher.
