# Supplementary material for: Risk of dementia or cognitive impairment in non-alcoholic fatty liver disease: A systematic review and meta-analysis
Source: Front Aging Neurosci. 2022 Sep 20;14:985109. doi: 10.3389/fnagi.2022.985109 (PMC9530447; doi:10.3389/fnagi.2022.985109)
Supplement: Supplementary file 1 [file Data_Sheet_1.doc]

**Appendix 1**

**Details of the Literature Search Strategy**

(1) PubMed

| **Search** | **Query** | **Items found** |
| --- | --- | --- |
| #1 | "Non-alcoholic Fatty Liver Disease"[Mesh] | 19193 |
| #2 | (((Non alcoholic Fatty Liver Disease[Title/Abstract]) OR (NAFLD[Title/Abstract])) OR (Nonalcoholic Fatty Liver*[Title/Abstract])) OR (Nonalcoholic Steatohepatiti*[Title/Abstract]) | 30823 |
| #3 | #1OR#2 | 33373 |
| #4 | (("Dementia"[Mesh]) OR "Alzheimer Disease"[Mesh]) OR "Cognitive Dysfunction"[Mesh] | 210989 |
| #5 | (((((((((((((dementia*[Title/Abstract]) OR (Amentia*[Title/Abstract])) OR (Alzheimer Dementia*[Title/Abstract])) OR (Alzheimer's Disease*[Title/Abstract])) OR (Senile Dementia*[Title/Abstract])) OR (Alzheimer Type Dementia*[Title/Abstract])) OR (Alzheimer Sclerosis[Title/Abstract])) OR (Alzheimer Syndrome[Title/Abstract])) OR (Alzheimer Disease*[Title/Abstract])) OR (Cognitive Dysfunction*[Title/Abstract])) OR (Cognitive Impairment*[Title/Abstract])) OR (Neurocognitive Disorder*[Title/Abstract])) OR (Cognitive Decline*[Title/Abstract])) OR (Mental Deterioration*[Title/Abstract]) | 312131 |
| #6 | #4 OR #5 | 357624 |
| #7 | #3 AND #6 | 160 |

(2) Embase

| **Search** | **Query** | **Items found** |
| --- | --- | --- |
| #1 | 'nonalcoholic fatty liver'/exp | 57209 |
| #2 | 'non alcoholic fatty liver disease':ab,ti OR nafld:ab,ti OR 'nonalcoholic fatty liver':ab,ti OR 'nonalcoholic steatohepatiti*':ab,ti | 48988 |
| #3 | #1 OR #2 | 64127 |
| #4 | 'dementia'/exp | 408756 |
| #5 | 'alzheimer disease'/exp | 225806 |
| #6 | 'cognitive defect'/exp | 562739 |
| #7 | dementia*:ab,ti OR amentia*:ab,ti OR 'alzheimer dementia*':ab,ti OR 'alzheimers disease*':ab,ti OR 'senile dementia*':ab,ti OR 'alzheimer type dementia*':ab,ti OR 'alzheimer sclerosis':ab,ti OR 'alzheimer syndrome':ab,ti OR 'alzheimer disease*':ab,ti OR 'cognitive dysfunction*':ab,ti OR 'cognitive impairment*':ab,ti OR 'neurocognitive disorder*':ab,ti OR 'cognitive decline*':ab,ti OR 'mental deterioration*':ab,ti | 322736 |
| #8 | #4 OR #5 OR #6 OR #7 | 613960 |
| #10 | #3 AND #8 | 708 |

(3) Cochrane Library

| **Search** | **Query** | **Items found** |
| --- | --- | --- |
| #1 | MeSH descriptor: [Non-alcoholic Fatty Liver Disease] explode all trees | 1253 |
| #2 | (Non alcoholic Fatty Liver Disease):ti,ab,kw OR (NAFLD):ti,ab,kw OR (Nonalcoholic Fatty Liver*):ti,ab,kw OR (Nonalcoholic Steatohepatiti*):ti,ab,kw | 3878 |
| #3 | #1 OR #2 | 3878 |
| #4 | MeSH descriptor: [Dementia] explode all trees | 6621 |
| #5 | MeSH descriptor: [Alzheimer Disease] explode all trees | 3732 |
| #6 | MeSH descriptor: [Cognitive Dysfunction] explode all trees | 2183 |
| #7 | (dementia*):ti,ab,kw OR (Amentia*):ti,ab,kw OR (Alzheimer Dementia*):ti,ab,kw OR (Alzheimer's Disease*):ti,ab,kw OR (Senile Dementia*):ti,ab,kw | 21449 |
| #8 | (Alzheimer Type Dementia*):ti,ab,kw OR (Alzheimer Sclerosis):ti,ab,kw OR (Alzheimer Syndrome):ti,ab,kw OR (Alzheimer Disease*):ti,ab,kw OR (Cognitive Dysfunction*):ti,ab,kw | 18408 |
| #9 | (Cognitive Impairment*):ti,ab,kw OR (Neurocognitive Disorder*):ti,ab,kw OR (Cognitive Decline*):ti,ab,kw OR (Mental Deterioration*):ti,ab,kw | 21455 |
| #10 | #4 OR #5 OR #6 OR #7 OR #8 OR #9 | 40609 |
| #11 | #3 AND #10 | 8 |

(4) [Web of Science](https://apps.webofknowledge.com/home.do?SID=6BQQjiiMCVa9MgFvRpC) core collection

| **Search** | **Query** | **Items found** |
| --- | --- | --- |
| #1 | **(((TS=(Non alcoholic Fatty Liver Disease)) OR TS=(NAFLD)) OR TS=(Nonalcoholic Fatty Liver*)) OR TS=(Nonalcoholic Steatohepatiti*)** | 42669 |
| #2 | **(((((((((((((TS=(dementia*)) OR TS=(Amentia*)) OR TS=(Alzheimer Dementia*)) OR TS=(Alzheimer's Disease*)) OR TS=(Senile Dementia*)) OR TS=(Alzheimer Type Dementia*)) OR TS=(Alzheimer Sclerosis)) OR TS=(Alzheimer Syndrome)) OR TS=(Alzheimer Disease*)) OR TS=(Cognitive Dysfunction*)) OR TS=(Cognitive Impairment*)) OR TS=(Neurocognitive Disorder*)) OR TS=(Cognitive Decline*)) OR TS=(Mental Deterioration*)** | 456706 |
| #3 | #1 AND #2 | 219 |
